# Clinical impact of initial surgical aortic bioprosthetic valve size on mid-term outcomes of transcatheter valve-in-valve in Japanese patients

**DOI:** 10.1007/s11748-026-02275-x

**Published:** 2026-02-27

**Authors:** Koichi Maeda, Satoru Domoto, Kei Torikai, Joji Ito, Yasuhiro Ichibori, Tohru Takaseya, Takeshi Onohara, Junji Yunoki, Hiromichi Sonoda, Hidenori Yoshitaka

**Affiliations:** 1 Department of Cardiovascular Surgery, The University of Osaka, 2-2 Yamada-Oka, Suita, Osaka 565-0871 Japan; 2 Department of Cardiovascular Surgery, Tokyo Women’s Medical University, Tokyo, Japan; 3 Department of Cardiovascular Surgery, Dokkyo Medical University Saitama Medical Center, Koshigaya, Japan; 4 Department of Cardiovascular Surgery, Tokyo Bay Urayasu Ichikawa Medical Center, Urayasu, Japan; 5 Department of Cardiology, Osaka Police Hospital, Osaka, Japan; 6 Department of Cardiovascular Surgery, Kurume University, Kurume, Japan; 7 Department of Cardiovascular Surgery, Tottori University, Tottori, Japan; 8 Department of Cardiovascular Surgery, Saga University, Saga, Japan; 9 Department of Cardiovascular Surgery, Kyusyu University, Fukuoka, Japan; 10 Department of Cardiovascular Surgery, The Sakakibara Heart Institute of Okayama, Okayama, Japan

**Keywords:** Transcatheter aortic valve in surgical aortic valve, Small bioprosthetic valve, Patient prosthesis mismatch

## Abstract

**Objective:**

Clinical outcomes of transcatheter aortic valve-in-surgical aortic valve (TAV-in-SAV) procedures have been reported to be poor in patients initially implanted with small bioprosthetic valves. The aim of this study was to evaluate mid-term outcomes of TAV-in-SAV for small SAVs using data from a Japanese multicenter registry.

**Methods:**

A total of 185 TAV-in-SAV procedures performed between January 2013 and March 2024 at 10 centers in Japan were analyzed. The primary endpoint was overall 4-year Kaplan–Meier survival. Clinical outcomes were also compared between initial ≤ 21 mm SAV (small group) vs. > 21 mm SAV (non-small group). The mean age was 82.4 years; 46.5% were female. One hundred sixteen (62.7%) patients were in the small group (≤ 21 mm SAV in 40 patients; > 21 mm SAV in 76 patients). The Evolut platform was used in 150 patients (81.1%), especially in the small group (103 patients, 88.8%).

**Results:**

The 4-year survival in the small group (69.1%) was comparable to that in the non-small group (79.6%) (*p* = 0.79). Similarly, freedom from cardiovascular mortality at 4 years in the small group (91.3%) was comparable to that in the non-small group (90.1%) (*p* = 0.096).

**Conclusion:**

This multicenter retrospective study shows that TAV-in-SAV in Japanese patients yields satisfactory mid-term outcomes. Despite advanced age, overall and freedom from cardiovascular mortality at 4 years were comparable to previous international reports, with no significant difference by valve size. (ClinicalTrials.gov number, NCT06826027.)

**Supplementary Information:**

The online version contains supplementary material available at 10.1007/s11748-026-02275-x.

## Introduction

Transcatheter aortic valve replacement (TAVR) has emerged as a treatment option for aortic valve replacement, and following numerous randomized trials, its indications have expanded from high- to low-risk patients. Conversely, a newer transcatheter technique for failed aortic bioprosthetic valves, transcatheter aortic valve-in-surgical aortic valve (TAV-in-SAV), has been performed since the early days of TAVR dissemination as a treatment for failed surgical bioprosthetic valves [1, 2]. This approach has shown relatively favorable mid-term outcomes in high-risk patients who are ineligible for conventional open-heart surgery [1–6]. However, outcomes of TAV-in-SAV have been reported to be poor in patients with stenotic prosthetic valve dysfunction or in those initially implanted with small bioprosthetic valves (≤ 21 mm)^2^. Because Japanese patients are generally of smaller stature and therefore more likely to receive bioprosthetic valves of 21 mm or less, mid-term outcomes of TAV-in-SAV in this population remain unclear. The objective of this study was to evaluate the mid-term outcomes of TAV-in-SAV in Japanese patients.

## Methods

### Patient

A total of 185 TAV-in-SAV procedures performed between January 2013 and March 2024 at 10 centers in Japan were included. The study protocol was developed in accordance with the Declaration of Helsinki and was approved by the ethics committee of each participating hospital. All patients provided written informed consent prior to participation in this study. Echocardiographic data were collected at the following time points: (1) baseline prior to TAV-in-SAV; and (2) 6 months post-procedure (6 ± 3 months after TAV-in-SAV]). Left ventricular ejection fraction (EF), peak aortic jet velocity, mean aortic valve gradient, aortic valve area, grade of valve regurgitation, effective orifice area (EOA), and prosthesis–patient mismatch (PPM) were assessed by echocardiography. EOA was calculated as the stroke volume of the left ventricular outflow tract divided by the velocity–time integral of the aortic jet and indexed to body surface area (BSA). PPM severity was classified by indexed EOA as none (≥ 0.85 cm^2^/m^2^), moderate (≥ 0.65 and < 0.85 cm^2^/m^2^), or severe (< 0.65 cm^2^/m^2^). Preoperative screening for TAV-in-SAV was performed by each facility’s Heart team, primarily using CT and echocardiography, to determine the type and size of the TAV, the approach site, and to assess the risk of coronary artery occlusion after TAV-in-SAV. Antiplatelet therapy and anticoagulant therapy following TAV-in-SAV were administered at the discretion of the attending physician at each participating hospital. Any event, including cardiovascular mortality or stroke, was defined according to VARC 3 criteria [7].

### Statistical analysis

Continuous variables are presented as mean ± standard deviation or median (interquartile range) and categorical variables as frequencies (%). Normality of continuous variable distributions was tested using the Shapiro–Wilk test, and data were analyzed accordingly. Paired data were assessed using a paired *t*-test for normally distributed variables and a Wilcoxon signed-rank test for non-normally distributed variables. Categorical variables were compared across independent groups using the chi-square test. For normally distributed continuous variables across independent groups, an independent samples *t*-test was used; for non-normally distributed variables, a Mann–Whitney U test was applied. Kaplan–Meier estimates were used to assess time-to-event variables, with comparisons by the log-rank test. All analyses were performed using JMP® (version 18.2.1, SAS Institute Inc., Cary, NC, USA).

The primary endpoint was overall 4-year survival, estimated using the Kaplan–Meier method. Secondary endpoints included freedom from cardiovascular mortality and from major adverse cardiovascular and cerebrovascular events (MACCE), also estimated using Kaplan–Meier and Cox proportional hazards modeling to identify mortality-associated factors. Comparisons were also made between initial SAV label sizes (≤ 21 mm label size [small group] vs. > 21 mm label size [non-small group]) and valve types (self-expanding vs. balloon-expandable) regarding hemodynamics, mortality, and MACCE. Propensity-score matching (1:1 matching, with the nearest neighbor matching without replacement) was performed to adjust for significant differences in baseline covariates and potential confounders that may lead to biased estimates between two arms. A propensity score for each patient was calculated by performing a nonparsimonious multivariable logistic regression. At baseline, we examined the following factors that showed significant differences and could potentially affect prognosis, excluding body mass index (BMI) and BSA: age, sex, chronic obstructive pulmonary disease (COPD), mechanism of surgical valve failure, preoperative mean pressure gradient, and pre indexed effective orifice area (iEOA). The Kaplan–Meier method was also used to examine overall 4-year survival and freedom from cardiovascular mortality within these groups after propensity-score maching.

The study was approved by the Ethics Committee of the University of Osaka (approval number: 23539).

## Results

Table 1 presents the clinical characteristics of the 185 patients in this registry. The mean age was 82.4 ± 5.6 years (range, 61–97), 46.4% were female, and the STS score averaged 11.4 ± 1.1 (range, 1.1–86.5). Surgical bioprosthetic valve dysfunction was due to stenosis in 83 patients (44.9%), regurgitation in 50 (27.0%), and combined dysfunction in 52 (28.1%). One hundred sixteen patients (62.7%) who had undergone surgical aortic valve replacement with ≤ 21 mm surgical valve group (small group) were predominantly female (84.9% vs. 15.1%, *p* < 0.0001), had smaller BSA (1.44 ± 0.15 vs. 1.60 ± 0.15 m^2^, *p* < 0.0001), BMI (21.4 ± 3.1 vs. 22.5 ± 2.8 kg/m^2^, *p* = 0.013), and a higher prevalence of stenotic failure mechanisms (55.2% vs. 27.5%, *p* = 0.0004) compared to those with > 21 mm surgical valve group (non-small group).

**Table 1:** Baseline characteristics at the time of TAV-in-SAV procedure

	All (*n* = 185)	≤ 21 mm (*n* = 116)	> 21 mm (*n* = 69)	*P* value
Age, mean (SD), y	82.4 (5.6)	83.5 (5.1)	80.5 (5.8)	0.0003
Women, no. (%)	86 (46.4)	73 (84.9)	13 (15.1)	< 0.0001
BSA, mean (SD), m^2^	1.50 (0.17)	1.44 (0.15)	1.60 (0.15)	< 0.0001
BMI, kg/m^2^	21.8 (3.0)	21.4 (3.1)	22.5 (2.8)	0.0130
STS PROM, mean (SD), %	11.4 (10.5)	12.2 (11.0)	10.1 (9.4)	0.19
Diabetes mellitus, no. (%)	29 (15.7)	15 (12.9)	14 (20.3)	0.21
IDDM, no. (%)	5 (2.7)	2 (1.7)	3 (4.4)	0.36
Peripheral vascular disease, no. (%)	12 (6.5)	7 (6.0)	5 (7.3)	0.76
Chronic renal failure (eGFR < 60 ml/min/1.73m^2^), no. (%)	27 (14.6)	16 (13.8)	11 (15.9)	0.67
Coronary artery disease, no. (%)	40 (21.6)	25 (21.6)	15 (21.7)	1.0
Cerebrovascular disease, no. (%)	21 (11.4)	12 (10.3)	9 (13.0)	0.63
COPD, no. (%)	51 (27.9)	25 (21.7)	26 (38.2)	0.026
Chronic AF, no. (%)	35 (18.9)	20 (17.2)	15 (21.7)	0.45
NYHA ≥ III, no. (%)	83 (44.9)	51 (44.0)	32 (46.4)	0.76
LVEF, mean (SD), %	57.3 (13.4)	57.7 (13.4)	56.6 (13.6)	0.60
*Surgical valve characteristics*				
Time since SAVR, mean (SD), y	10.6 (4.7)	10.6 (4.3)	10.5 (5.2)	0.88
Stentless, no. (%)	5 (2.7)	0 (0)	5 (7.3)	0.0066
*Surgical valve size*				
19 mm	40 (21.6)	40 (34.5)	(0)	
21 mm	76 (41.1)	76 (65.5)	(0)	
23 mm	46 (24.9)	0 (0)	46 (66.7)	
25 mm	22 (11.9)	0 (0)	22 (31.9)	
27 mm	1 (0.5)	0 (0)	1 (1.4)	
Mechanism of surgical valve failure				0.0004
Stenosis, no. (%)	83 (44.9)	64 (55.2)	19 (27.5)	
Regurgitation, no. (%)	50 (27.0)	22 (19.0)	28 (40.6)	
Combined, no. (%)	52 (28.1)	30 (25.9)	22 (31.9)	
Aortic valve regurgitation ≥ moderate at baseline, no. (%)	91 (49.2)	47 (40.5)	44 (63.8)	0.0025
Aortic valve mean gradient at baseline, mean (SD), mmHg	33.4 (15.1)	36.0 (14.9)	29.0 (14.7)	0.0024
Aortic valve area at baseline, mean (SD), cm^2^	0.99 (0.80)	0.81 (0.30)	1.28 (1.21)	0.0001
iEOA at baseline, mean (SD), cm^2^/m^2^	0.65 (0.45)	0.57 (0.21)	0.79 (0.66)	0.0010

SD, standard deviation; BSA, body surface area; BMI, body mass index; STS PROM, the society of thoracic surgeons predicted risk of mortality score; IDDM, insulin-dependent diabetes mellitus; COPD, chronic obstructive pulmonary disease; NYHA, New York Heart Association; LVEF, left ventricular ejection fraction; SAVR, surgical aortic valve replacement; iEOA, indexed effective orifice area

Table 2 shows early clinical outcomes. The Evolut platform was used in 150 patients (81.1%), especially in the small group (88.8%) (95.0% in < 21 mm SAV, 85.5% in > 21 mm SAV); in contrast, the SAPIEN platform was more common with larger valve sizes (≥ 23 mm). A total of 167 patients (90.3%) underwent the transfemoral approach, with a mean procedural time of 79.7 min. Thirty-day mortality occurred in 2 patients (1.1%)—1 due to left ventricular rupture and another due to aortic dissection. There were 2 disabling strokes (1.1%) and 8 access-related complications (4.3%), without differences between the groups.

**Table 2:** Early clinical outcomes

	All (*n* = 185)	≤ 21 mm (*n* = 116)	> 21 mm (*n* = 69)	*P* value
*Device used*				
Evolut platform, no. (%)	150 (81.1)	103 (88.8)	47 (68.1)	
23 mm, no. (%)	110 (59.5)	101 (87.1)	9 (13.0)	< 0.0001
26 mm, no. (%)	37 (20.0)	1 (0.9)	36 (52.2)	
29 mm, no. (%)	3 (1.6)	1 (0.9)	2 (2.9)	
SAPIEN platform, no. (%)	35 (18.9)	13 (11.2)	22 (31.9)	
20 mm, no. (%)	7 (3.8)	7 (6.0)	0 (0)	< 0.0001
23 mm, no. (%)	19 (10.3)	6 (5.2)	13 (18.9)	
26 mm, no. (%)	8 (4.3)	0 (0)	8 (11.6)	
29 mm, no. (%)	1 (0.5)	0 (0)	1 (1.4)	
Approach				0.26
Transfemoral, no. (%)	167 (90.3)	104 (89.7)	63 (91.3)	
Transapical, no. (%)	9 (4.9)	4 (3.5)	5 (7.3)	
Transaortic, no. (%)	1 (0.5)	1 (0.9)	0 (0)	
Transsubclavian, no. (%)	8 (4.3)	7 (6.0)	1 (1.5)	
Concomitant procedure, no. (%)	6 (3.2)	4 (3.5)	2 (2.9)	1.0
PCI, no. (%)	3 (1.6)	3 (2.9)	0 (0)	0.29
TEVAR, no. (%)	3 (1.6)	1 (0.9)	2 (2.9)	0.56
Operating time, mean (SD), min	79.7 (34.4)	79.6 (34.2)	80.0 (34.9)	0.95
No transfusion, no. (%)	139 (75.1)	92 (79.3)	47 (68.1)	0.11
Duration of ICU stay, median (IQR), d	2 (2–2)	2 (2–2)	2 (2–2)	0.62
Duration of postoperative hospital stay, median (IQR), d	8 (5–11)	7.5 (5–10)	9 (5–12)	0.15
*30-day outcomes*				
Death, no. (%)	2 (1.1)	2 (1.7)	0 (0)	0.53
Cardiovascular death, no. (%)	1 (0.5)	1 (0.9)	0 (0)	1.0
Disabling stroke, (SD), %	2 (1.1)	1 (0.9)	1 (1.5)	1.0
Major access-related complication, no. (%)	8 (4.3)	3 (2.6)	5 (7.3)	0.15
Major/life-threatening bleeding, no. (%)	5 (2.7)	2 (1.7)	3 (4.4)	0.36
New Permanent pacemaker implantation, no. (%)	2 (1.2)	0 (0)	2 (3.5)	0.13

SD, standard deviation; PCI, percutaneous coronary intervention; TEVAR, thoracic endovascular aortic repair; ICU, intensive care unit; IQR, interquartile range

At 6 months (Table 3), residual aortic regurgitation was less than trace in 86.9% of cases. The small group had higher peak velocity, higher mean gradient, and smaller indexed EOA, and severe PPM frequently occurred in the small group (25.0% vs. 12.3% in the non-small group; *p* = 0.052). However, the Evolut platform showed a trend toward better postoperative hemodynamics, including lower gradients and reduced PPM incidence, compared to the SAPIEN platform (Supplementary Table 1 and Supplementary Fig. 1). The Evolut platform demonstrated a low incidence of severe PPM regardless of SAV size, whereas the SAPIEN platform showed a higher incidence with smaller SAV sizes (Supplementary Fig. 2).

**Table 3:** Echocardiographic findings at 6 months post-procedure

	All (*n* = 175)	≤ 21 mm (*n* = 110)	> 21 mm (*n* = 65)	
Aortic regurgitation				0.73
None, no. (%)	116 (66.3)	74 (67.3)	42 (64.6)	
Trace, no. (%)	36 (20.6)	21 (19.1)	15 (23.1)	
Mild, no. (%)	22 (12.6)	14 (12.7)	8 (12.3)	
≥ Moderate, no. (%)	1 (0.6)	1 (0.9)	0 (0)	
Peak velocity, mean (SD), m/s	2.4 (0.6)	2.5 (0.6)	2.2 (0.6)	0.0036
Aortic valve gradient, mean (SD), mmHg	13.1 (6.5)	14.2 (6.5)	11.3 (6.0)	0.0037
Aortic valve area, mean (SD), cm^2^	1.32 (0.42)	1.21 (0.40)	1.50 (0.39)	< 0.0001
iEOA, mean (SD), cm^2^/m^2^	0.89 (0.28)	0.86 (0.30)	0.95 (0.24)	0.0421
*PPM*				
≥ Moderate PPM, no. (%)	78 (45.1)	57 (52.8)	21 (32.3)	0.012
Evolut platform, no. (%)	58 (41.1)	45 (47.4)	13 (28.3)	0.044
SAPIEN platform, no (%)	20 (62.5)	12 (92.3)	8 (42.1)	0.0079
Severe PPM, no. (%)	35 (20.2)	27 (25.0)	8 (12.3)	0.052
Evolut platform, no. (%)	25 (17.7)	19 (20.0)	6 (13.0)	0.036
SAPIEN platform, no (%)	10 (31.3)	8 (61.5)	2 (10.5)	0.0051

SD, standard deviation; iEOA, indexed effective orifice area; PPM, patient-prosthesis mismatch

**Fig. 1:**
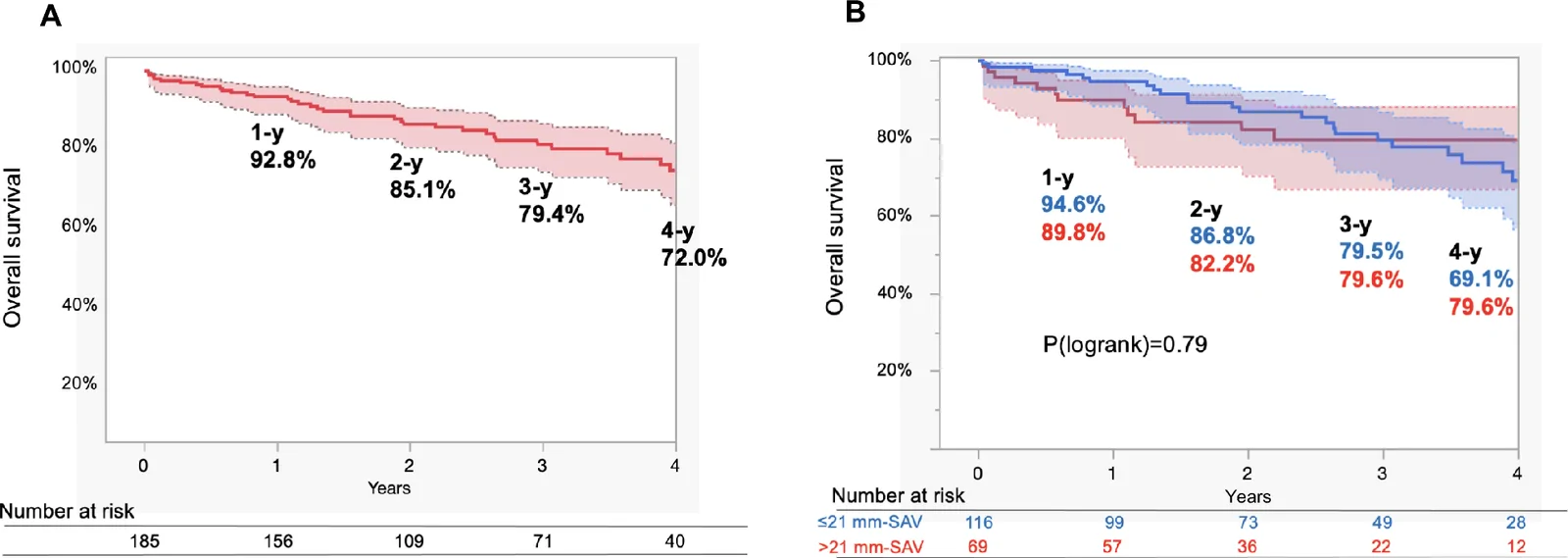
Kaplan–Meier analysis of overall survival

**Fig. 2:**
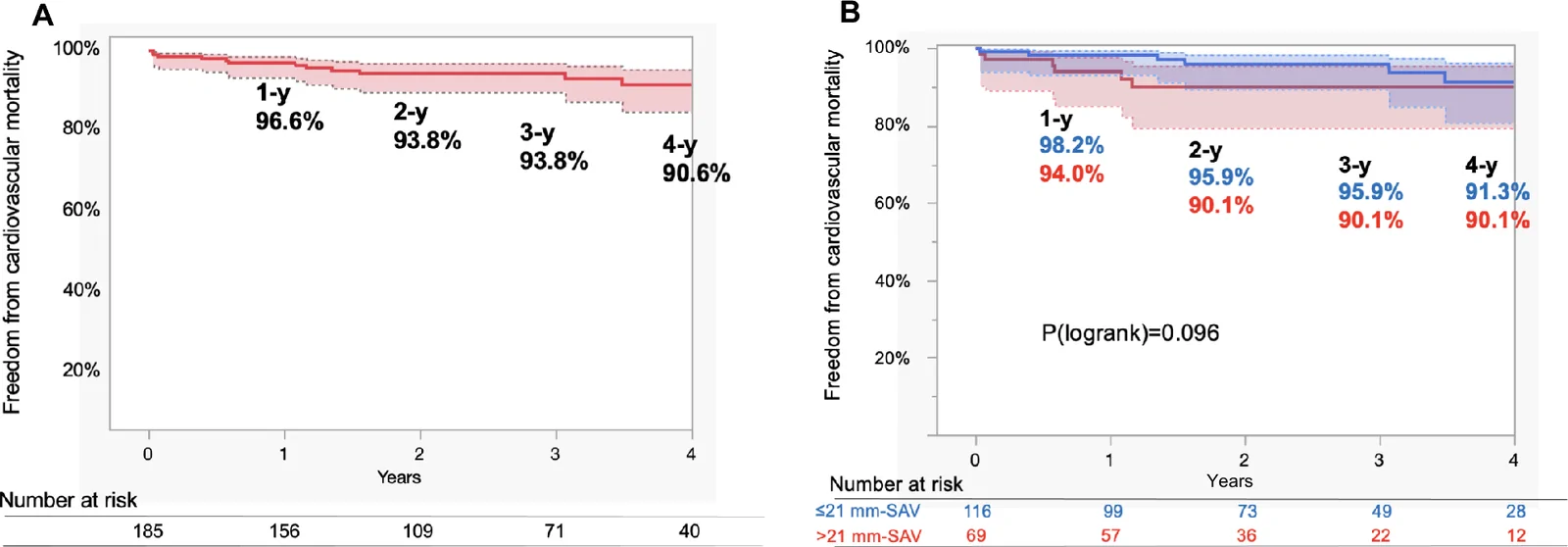
Kaplan–Meier analysis of freedom from cardiovascular mortality CV, cardiovascular

**Fig. 3:**
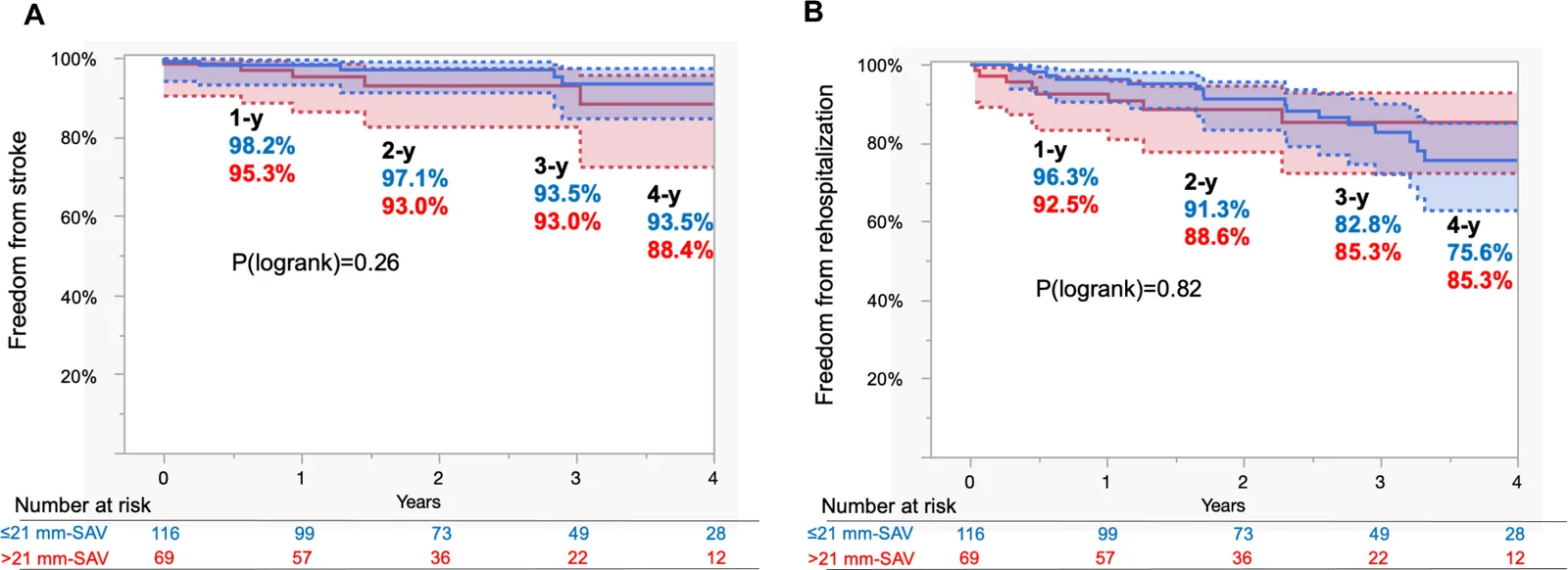
Kaplan–Meier analysis of freedom from Stroke (3A) and freedom from rehospitalization due to cardiac events (3B) SAV, surgical aortic valve

**Fig. 4:**
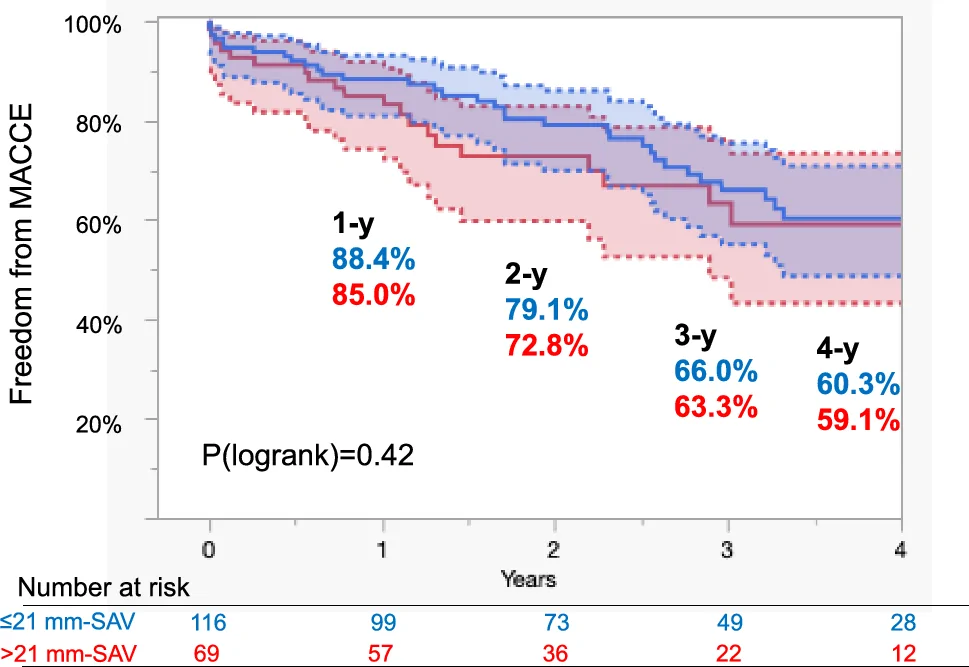
Kaplan–Meier analysis of freedom from MACCE MACCE, major adverse cardiovascular and cerebrovascular events

Mean follow-up was 2.6 ± 1.7 years (maximum, 7.7 years). There were 39 deaths: 10 respiratory-related, 4 from cancer, and 10 of unknown cause, including 4 sudden deaths (Supplementary Table 2). Overall survival rates were 92.8% at 1 year, 85.1% at 2 years, 79.4% at 3 years, and 72.0% at 4 years (Fig. 1A). Freedom from cardiovascular mortality was 96.6%, 93.8%, 93.8%, and 90.6% at 1 through 4 years, respectively (Fig. 2A). There were no significant differences in overall survival or freedom from cardiovascular mortality between the groups (Figs. 1B, 2B). These trends yielded similar results even in well-matched patient cohorts (Supplementary Table 3 and Supplentary Fig. 3A, B). Furthermore, no significant differences were observed in these outcomes between the 19 mm valve and the ≥ 21 mm valve (Supplementary Fig. 4A, B). Multivariate Cox analysis identified age as an independent predictor of all-cause death (HR 1.09, *P* = 0.0083) (Table 4). Freedom from Stroke (Fig. 3A) and freedom from rehospitalization due to cardiac events (Fig. 3B) were not associated with valve size. As a result, no difference was observed between the two groups regarding freedom from MACCE (Fig. 4). Furthermore, no difference was observed in these results when comparing the 19 mm valve with the > 19 mm valve (Supplementary Fig. 5A–C).

**Table 4:** Uni- and multivariable analysis for all-cause mortality

	Univariate analysis			Multivariate analysis		
	HR	95% CI	*P*-value	HR	95% CI	*P*-value
Age (per 1-year increments)	1.08	1.02–1.16	0.012	1.09	1.02–1.16	0.0083
Male	1.53	0.82–2.87	0.18			
Pre-EF < 50%	1.82	0.98–3.36	0.058	1.64	0.89–3.06	0.210
Chronic AF	1.55	0.73–3.26	0.25			
NYHA ≥ III	1.77	0.97–3.23	0.065	1.61	0.87–2.97	0.13
≤ 21 mm-SAV	0.91	0.49–1.73	0.79			
Use of Evolut platform	0.58	0.30–1.14	0.12	0.55	0.28–1.08	0.080

EF left ventricular ejection fraction, AF atrial fibrillation, NYHA New York Heart Association, SAV surgical aortic valve

Bioprosthetic valve thrombosis occurred in 10 patients (5.4%), with no significant difference between the groups (≤ 21 mm 3.5% vs. > 21 mm 8.7%, *P* = 0.18) or THV types (Evolut 4.7% vs. SAPIEN 8.6%, *P* = 0.40). Incidence was unrelated to antiplatelet therapy at discharge (*P* = 0.69) but tended to be lower with anticoagulant therapy (*P* = 0.058) (Supplementary Table 4).

## Discussion

This study presents the mid-term results of TAV implantation for SAV with prosthetic valve dysfunction in Japanese patients. In the small group, the outcomes of narrow bioprosthetic valves, such as ≤ 21 mm or > 21 mm, were comparable to those of larger prosthetic valves. The mid- to long-term outcomes were also similar to those in previous reports, despite the patients in this study being older, with a mean age of 82.4 years, compared to most prior cohorts in their 70s^1,3,4,6^. The overall survival rate was 72.0% at 4 years, and cardiovascular survival was 90.6%, which is favorable; however, deaths from respiratory disease, including pneumonia, accounted for about one-quarter of all deaths, and the effect of age on these results cannot be excluded.

The present findings differ from those of earlier studies, suggesting a worse prognosis for TAV-in-SAV in small SAVs [1.2], likely owing to differences in body size. In this study, the mean body surface area was 1.50 m^2^, compared with 1.85 m^2^ in prior reports. Hemodynamic parameters in the small group were slightly worse compared to the non-small group, but the Evolut platform provided stable hemodynamics regardless of SAV valve size. In this cohort, 81.1% of patients received the Evolut platform, including 88.8% of those with ≤ 21 mm and 95% of those with 19 mm bioprosthetic valves. The SAPIEN platform was used more often for larger bioprosthetic valves. Although the Evolut platform showed better hemodynamic performance, such as lower PPM rates and mean gradients, there was no observed correlation with prognosis. This may be because the Evolut was predominantly used for small valves and the SAPIEN for large valves. The Evolut platform was effective for narrow valve annuli.

Likewise, there were no differences in long-term prognosis or freedom from MACCE, including stroke and rehospitalization, over 4 years. These results suggest that mid-term prognosis can be maintained if the bioprosthetic valve is appropriately sized for the patient's body, even when it is ≤ 21 mm or > 21 mm at the initial SAVR. Therefore, it is not essential to implant an SAV that exceeds the size appropriate for the patient's physique using a root enlargement procedure with a Y-shaped incision [8].

Clinical valve thrombus occurred in 5.4% of patients. While various trials have examined antiplatelet and anticoagulant therapy after TAVR, few have reached final results, and solid evidence remains lacking [9–11]. The effect of anticoagulation after TAV-in-SAV has not been reported, but Japanese TAVR patients are generally considered to have a high bleeding risk [12]. The long-term effects of thrombus are debated; further research on optimal postoperative anticoagulation is warranted.

In routine practice, redo-SAVR is often preferred for patients with few comorbidities and a long life expectancy, whereas TAV-in-SAV is chosen for high-risk or frail patients. Over the past decade, improved surgical experience, perioperative management, and valve designs have increased the use of TAV-in-SAV in younger patients. A systematic review comparing redo-SAVR and TAV-in-SAV found that TAV-in-SAV had better early outcomes, while redo-SAVR was superior in the mid-term and beyond [5], highlighting the need for careful heart team discussion. No randomized controlled trials have been conducted to date, and further studies on redo-SAVR in Japanese AS patients are needed to evaluate mid-term valve-in-valve outcomes.

### Study limitations

This study has some limitations. First, this retrospective study involved a small cohort, limiting statistical power and significance, and the possibility of Type II error due to the small sample size cannot be ruled out. Second, the lack of an echocardiographic core laboratory may have introduced inter-institution variation in hemodynamic assessments, including PPM. Third, it is highly likely that a certain number of patients were excluded due to various anatomical limitations identified through preoperative screenig conducted at each heart team. Since CT data collection was not performed in this study, these anatomical risk factors such as valve to coronary distance and valve to aorta distance before and after TAV-in-SAV could not be obtained. Finally, ideally, cardiovascular death, stroke, and hospitalization due to heart failure should be adjudicated by a clinical events committee; however, this was not included in this study.

## Conclusion

This multicenter retrospective study demonstrates that TAV-in-SAV procedures in Japanese patients—particularly those with small bioprosthetic valves such as ≤ 21 or > 21 mm—yield satisfactory mid-term clinical outcomes. Despite the advanced age and small body size of this high-risk cohort, all-cause and cardiovascular survival rates at 4 years were comparable to previous international reports, with no significant differences by valve size.

## Supplementary Information

Below is the link to the electronic supplementary material.

Supplementary file1 (DOCX 679 KB)

## Data Availability

The Data will be shared with the corresponding authors upon reasonable request.
