# Supplementary material for: Clinical impact of initial surgical aortic bioprosthetic valve size on mid-term outcomes of transcatheter valve-in-valve in Japanese patients
Source: Gen Thorac Cardiovasc Surg. 2026 Feb 27;74(9):831–7. doi: 10.1007/s11748-026-02275-x (PMC13506638; doi:10.1007/s11748-026-02275-x)
Supplement: Supplementary file 1 [file 11748_2026_2275_MOESM1_ESM.docx]

**Clinical impact of initial surgical aortic bioprosthetic valve size on mid-term outcomes of transcatheter valve-in-valve in Japanese patients**

Journal: *General Thoracic and Cardiovascular Surgery*

Koichi Maeda, MD, PhD^a^; Satoru Domoto, MD, PhD^b^; Kei Torikai, MD, PhD^c^; Joji Ito, MD, PhD^d^; Yasuhiro Ichibori, MD, PhD^e^; Tohru Takaseya, MD; PhD^f^, Takeshi Onohara, MD, PhD^g^; Junji Yunoki, MD, PhD^h^; Hiromichi Sonoda, MD, PhD^i^, Hidenori Yoshitaka, MD^j^.

^a^ Department of Cardiovascular Surgery, The University of Osaka

^b^ Department of Cardiovascular Surgery, Tokyo Women’s Medical University

^c^ Department of Cardiovascular Surgery, Dokkyo Medical University Saitama Medical Center

^d^ Department of Cardiovascular Surgery, Tokyo Bay Urayasu Ichikawa Medical Center

^e^ Department of Cardiology, Osaka Police Hospital

^f^ Department of Cardiovascular Surgery, Kurume University

^g^ Department of Cardiovascular Surgery, Tottori University

^h^ Department of Cardiovascular Surgery, Saga University

^i^ Department of Cardiovascular Surgery, Kyusyu University

^j^ Department of Cardiovascular Surgery, The Sakakibara Heart Institute of Okayama

**Correspondence:** Koichi Maeda, MD, PhD

Department of Cardiovascular Surgery, The University of Osaka

2-2 Yamada-oka, Suita, Osaka 565-0871, Japan

Tel: +81-6-6879-3154; Fax: +81-6-6879-3163

E-mail: [k-maeda@surg1.med.osaka-u.ac.jp](mailto:k-maeda@surg1.med.osaka-u.ac.jp)

Electronic Supplementary Material

(Supplementary Table 1) Hemodynamic performance of Evolut and SAPIEN platform at 6-months postoperatively.

|  | All  (n=173) | Evolut platform  (n=150) | SAPIEN platform  (n=35) |  |
| --- | --- | --- | --- | --- |
| Peak velocity, mean (SD), m/s | 2.4(0.6) | 2.3(0.5) | 2.8(0.6) | <0.0001 |
| Aortic valve gradient, mean (SD), mmHg | 13.1(6.5) | 11.8 (5.3) | 18.9(8.0) | <0.0001 |
| Aortic valve area, mean (SD), cm^2^ | 1.32(0.42) | 1.20(0.37) | 1.35(0.42) | 0.074 |
| iEOA, mean (SD), cm^2^/m^2^ | 0.89(0.28) | 0.92(0.29) | 0.79(0.23) | 0.020 |

SD, standard deviation; iEOA, indexed effective orifice area.

(Supplementary Table 2) Reason for all-cause mortality

| The reason of death |  |
| --- | --- |
| Respirator-related, N (%) | 10 (25.6) |
| Cancer, N (%) | 4 (10.3) |
| sepsis, N (%) | 3 (7.7) |
| Heart failure, N (%) | 3 (7.7) |
| PVE, N (%) | 2 (5.1) |
| Bleeding, N (%) | 2 (5.1) |
| Stroke, N (%) | 2 (5.1) |
| MOF, N (%) | 2 (5.1) |
| AAD, N (%) | 1 (2.6) |
| Unknown, (%) | 10 (25.6) |

PVE, prosthetic valve endocarditis; MOF, multiple organ failure; AAD, acute aortic dissection

(Supplementary Table 3) Baseline characteristics at the time of TAV-in-SAV procedure after the propensity-score matching.

|  | All  (n=70) | ≤21mm  (n=35) | >21mm  (n=35) | P Value |
| --- | --- | --- | --- | --- |
| Age, mean (SD), y | 82.0 (5.6) | 82.3 (5.5) | 81.7 (5.7) | 0.66 |
| Women, No. (%) | 49 (70.0) | 25 (71.4) | 24 (68.6) | 1.0 |
| BSA, mean (SD), m^2^ | 1.53 (0.16) | 1.50 (0.14) | 1.56 (0.17) | 0.089 |
| BMI, kg/m^2^ | 21.8 (2.6) | 21.2 (2.9) | 22.4 (2.0) | 0.042 |
| STS PROM, mean (SD), % | 13.0 (13.6) | 14.4 (17.0) | 11.5 (8.9) | 0.39 |
| Diabetes mellitus, No. (%)  IDDM, No. (%) | 11 (15.7)  2 (2.9) | 5 (14.3)  1 (2.9) | 6 (17.1)  1 (2.9) | 1.0  1.0 |
| Peripheral vascular disease, No. (%) | 8 (11.4) | 5 (14.3) | 3 (8.6) | 0.71 |
| Chronic renal failure (eGFR <60 ml/min/1.73m^2^), No. (%) | 7 (10.0) | 3 (8.6) | 4 (11.4) | 0.67 |
| Coronary artery disease, No. (%) | 22 (31.4) | 12 (34.3) | 10 (28.6) | 0.80 |
| Cerebrovascular disease, No. (%) | 8 (11.4) | 3 (8.6) | 5 (14.3) | 0.71 |
| COPD, No. (%) | 25 (35.7) | 12 (34.3) | 13 (37.1) | 1.0 |
| Chronic AF, No. (%) | 15 (21.4) | 5 (14.3) | 10 (28.6) | 0.24 |
| NYHA ≥III, No. (%) | 38 (54.3) | 20 (57.1) | 18 (51.4) | 0.81 |
| LVEF, mean (SD), % | 55.2 (13.8) | 52.1 (13.7) | 58.3 (13.4) | 0.060 |
| Surgical valve characteristics | | | | |
| Surgical valve size  19 mm  21 mm  23 mm  25 mm  27 mm | 6 (8.6)  29 (41.4)  26 (37.1)  9 (12.9)  0 (0) | 6 (17.1)  29 (82.9)  0 (0)  0 (0)  0 (0) | (0)  (0)  26 (74.3)  9 (25.7)  0 (0) |  |
| Mechanism of surgical valve failure  Stenosis, No. (%)  Regurgitation, No. (%)  Combined, No. (%) | 27 (38.6)  20 (28.6)  23 (32.9) | 15 (42.9)  10 (28.6)  10 (28.6) | 12 (34.3)  10 (28.6)  13 (37.1) | 0.70 |
| Aortic valve regurgitation ≥moderate at baseline, No. (%) | 43 (61.4) | 21 (60.0) | 22 (62.9) | 1.0 |
| Aortic valve mean gradient at baseline, mean (SD), mmHg | 30.1 (12.9) | 28.9 (11.4) | 31.2 (14.3) | 0.45 |
| Aortic valve area at baseline, mean (SD), cm^2^ | 1.00 (0.40) | 0.98 (0.40) | 1.01 (0.41) | 0.75 |
| iEOA at baseline, mean (SD), cm^2^/m^2^ | 0.66 (0.27) | 0.66 (0.28) | 0.65 (0.27) | 0.91 |

SD, standard deviation; BSA, body surface area; BMI, body mass index; STS PROM, the society of thoracic surgeons predicted risk of mortality score; IDDM, insulin-dependent diabetes mellitus; COPD, chronic obstructive pulmonary disease; NYHA, New York Heart Association; LVEF, left ventricular ejection fraction; SAVR, surgical aortic valve replacement; iEOA, indexed effective orifice area.

(Supplementary Table 4) Incidence of valve thrombosis after TAV-in-SAV

|  | Valve thrombosis (+)  (n=10) | Valve thrombosis (-)  (n=175) |  |
| --- | --- | --- | --- |
| 19-mm SAV, N (%)  >19-mm SAV, N(%) | 0 (0)  10 (6.9) | 40 (22.9)  135 (77.1) | 0.12 |
| Evolut platform, N (%)  SAPIEN platform, N (%) | 7 (70.0)  3 (30.0) | 143 (81.7)  32 (18.3) | 0.40 |
| Antiplatelet therapy, N (%)  Non-antiplatelet therapy, N(%) | 9 (90.0)  1 (10.0) | 136 (77.7)  39 (22.3) | 0.69 |
| Anticoagulant therapy, N (%)  Non-anticoagulant therapy, N (%) | 2 (20.0)  8 (80.0) | 91 (52.0)  84 (48.0) | 0.058 |

FigS1

The Evolut platform showed a trend toward better postoperative hemodynamics, including lower gradients and reduced PPM incidence, compared to the SAPIEN platform.

PPM, patient-prosthesis mismatch

FigS2

The Evolut platform demonstrated a low incidence of severe PPM regardless of SAV size, whereas the SAPIEN platform showed a higher incidence with smaller SAV sizes.

PPM, patient-prosthesis mismatch; SAV, surgical aortic valve

FigS3

After propensity-score matching, there were no significant differences in overall survival overall survival (S3A) and freedom from cardiovascular mortality (S3B) between the groups.

CV, cardiovascular; SAV, surgical aortic valve

FigS4

No significant differences were observed in these outcomes between the 19 mm valve and the ≥21 mm valve

SAV, surgical aortic valve

FigS5

According to freedom from stroke, freedom from rehospitalization, and freedom from MACCE, no difference was observed when comparing the ≤21 mm valve with the >21 mm valve.

MACCE, major adverse cardiovascular and cerebrovascular events; SAV, surgical aortic valve.
